# Restoring the Flow: Observational Outcomes of Endoscopic Endonasal Dacryocystorhinostomy in a Tertiary Care Institution

**DOI:** 10.7759/cureus.92730

**Published:** 2025-09-19

**Authors:** Amarnath SB, Gouthami sushma S, Visweswara Rao Guthi

**Affiliations:** 1 Otolaryngology - Head and Neck Surgery, Sri Venkateswara Institute of Medical Sciences – Sri Padmavathi Medical College for Women, Tirupati, IND; 2 Community Medicine, Sri Venkateswara Institute of Medical Sciences – Sri Padmavathi Medical College for Women, Tirupati, IND

**Keywords:** chronic dacryocystitis, chronic epiphora, endonasal dcr, endoscopic dcr, nasolacrimal duct obstruction

## Abstract

Introduction: Nasolacrimal duct obstruction is a clinical condition characterized by epiphora and both acute and chronic dacryocystitis. Endoscopic endonasal dacryocystorhinostomy (EE DCR) is the treatment of choice for chronic dacryocystitis. This study aimed to evaluate the anatomical and functional success, safety profile, and patient-reported outcomes of EE DCR in chronic dacryocystitis in a tertiary care center.

Method: A retrospective review of 50 patients who underwent EE DCR between January 2020 and January 2025 was conducted. Data on demographics, clinical presentation, surgical technique, postoperative outcomes, and complications were gathered. Symptomatic improvement was measured using the Nasolacrimal Duct Obstruction Symptom Score (NLDO-SS), and quality of life was assessed using a validated questionnaire both preoperatively and postoperatively at six months. Statistical analysis was performed using IBM SPSS Statistics for Windows, Version 26 (Released 2019; IBM Corp., Armonk, New York).

Results: The mean age was 35.2 ± 13.3 years with a female predominance of 31 (62%). Epiphora was the chief complaint in 42 (84%) of cases. At six months, anatomical patency was achieved in 48 (96%), and subjective symptom resolution occurred in 49 (98%). Mean NLDO-SS and quality of life scores improved significantly (P < 0.001). Complications were reported in 6 patients (12%), including intraoperative hemorrhage in 2 (4%), postoperative adhesions in 2 (4%), and granulation tissue formation in 2 (4%). No vision-threatening events were recorded.

Conclusion: EE DCR demonstrates high anatomical and functional success with low complication rates, offering a minimally invasive and effective alternative to external DCR. The procedure yields substantial improvements in patient symptoms and quality of life. Further prospective studies with larger cohorts and longer follow-up periods are warranted to validate these findings.

## Introduction

Nasolacrimal duct obstruction is a clinical condition characterized by epiphora and both acute and chronic dacryocystitis, with surgical intervention being the definitive treatment of choice. Lacrimal surgery, akin to various other domains within medicine, has undergone substantial transformations over time, adapting to the evolving needs of patients and surgeons alike. Historically, conservative management was the prevailing approach for addressing this inflammation of the lacrimal sac; however, it frequently culminated in complications such as lacrimal abscesses, fistulas, and other morbidities that could significantly diminish the patient's quality of life [1].

Dacryocystorhinostomy (DCR) is the surgical procedure that facilitates the recreation of lacrimal flow into the nasal cavity by establishing an opening at the level of the lacrimal sac, thus circumventing the nasolacrimal duct. This surgery may be performed via either an external approach or an endonasal technique. Toti was the first to delineate the external DCR, while Caldwell articulated the endonasal method. With the progression of surgical techniques, endonasal DCR has emerged as the preferred treatment modality for chronic dacryocystitis. This minimally invasive procedure offers an effective means to mitigate these complications and boasts several advantages over traditional methodologies [2].

However, with the introduction of novel endoscopic techniques and advancements in camera technology, the field of endoscopic endonasal DCR (EE-DCR) has experienced a remarkable evolution. The benefits of EE-DCR extend beyond the provision of exceptional visualization of the nasal cavity, which allows the surgeon to meticulously access and address the affected region. This enhanced visibility translates into superior surgical precision and markedly improved patient outcomes [3,4]. By avoiding external incisions, the procedure minimizes scarring and cosmetic issues, reduces postoperative discomfort, and ensures smoother recovery. These benefits collectively improve surgical success, shorten hospital stays, and enhance overall treatment efficiency [5].

Notwithstanding its evident advantages, EE-DCR is not without its challenges, specifically the formidable learning curve associated with the procedure. Mastery necessitates specialized training and skill [6], which may require considerable time for surgeons to acquire. This study aims to analyze the anatomical and functional success, safety profile, and outcomes of endoscopic endonasal DCR in our tertiary care institution.

## Materials and methods

A total of 50 patients who presented from January 2020 to January 2025 to the Department of ENT at Sri Venkateswara Institute of Medical Sciences - Sri Padmavathi Medical College for Women, a tertiary care referral center located in Tirupati, Andhra Pradesh, India, exhibiting clinical evidence of established nasolacrimal duct obstruction and who underwent EE-DCR, were enrolled in this study. The study garnered approval from the Institutional Ethics Committee (IEC no. 1811).

Comprehensive case files of all patients encompassed in the study were meticulously retrieved, and information pertaining to clinical history, examination findings, and investigations, including diagnostic nasal endoscopy (DNE), X-ray of the paranasal sinuses (wherever indicated), and lacrimal syringing, were diligently compiled and reviewed alongside operative procedures, postoperative results, and complications.

**Figure 1:**
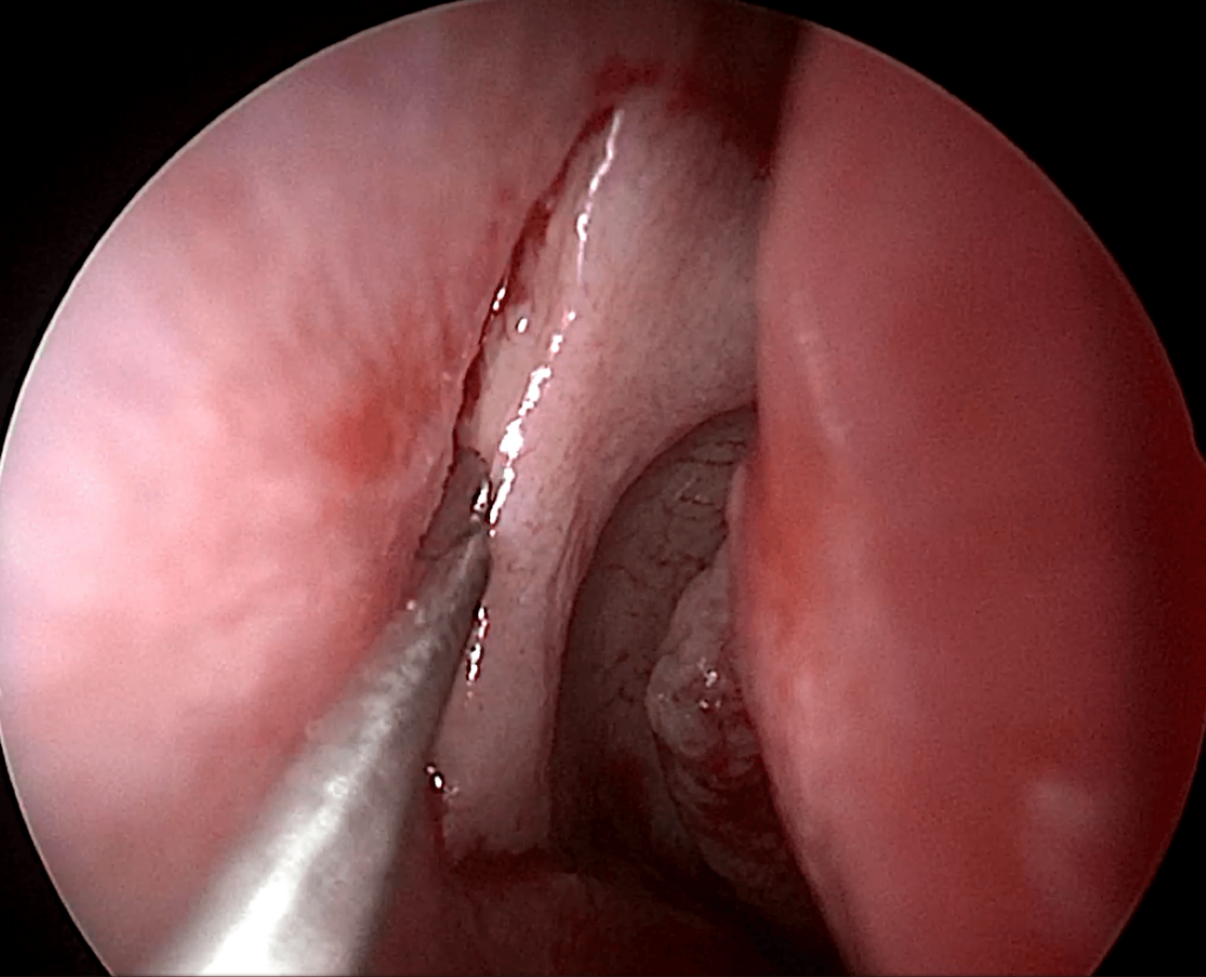
Endoscopic picture showing incisions for elevation of posteriorly based mucoperiosteal flap

**Figure 2:**
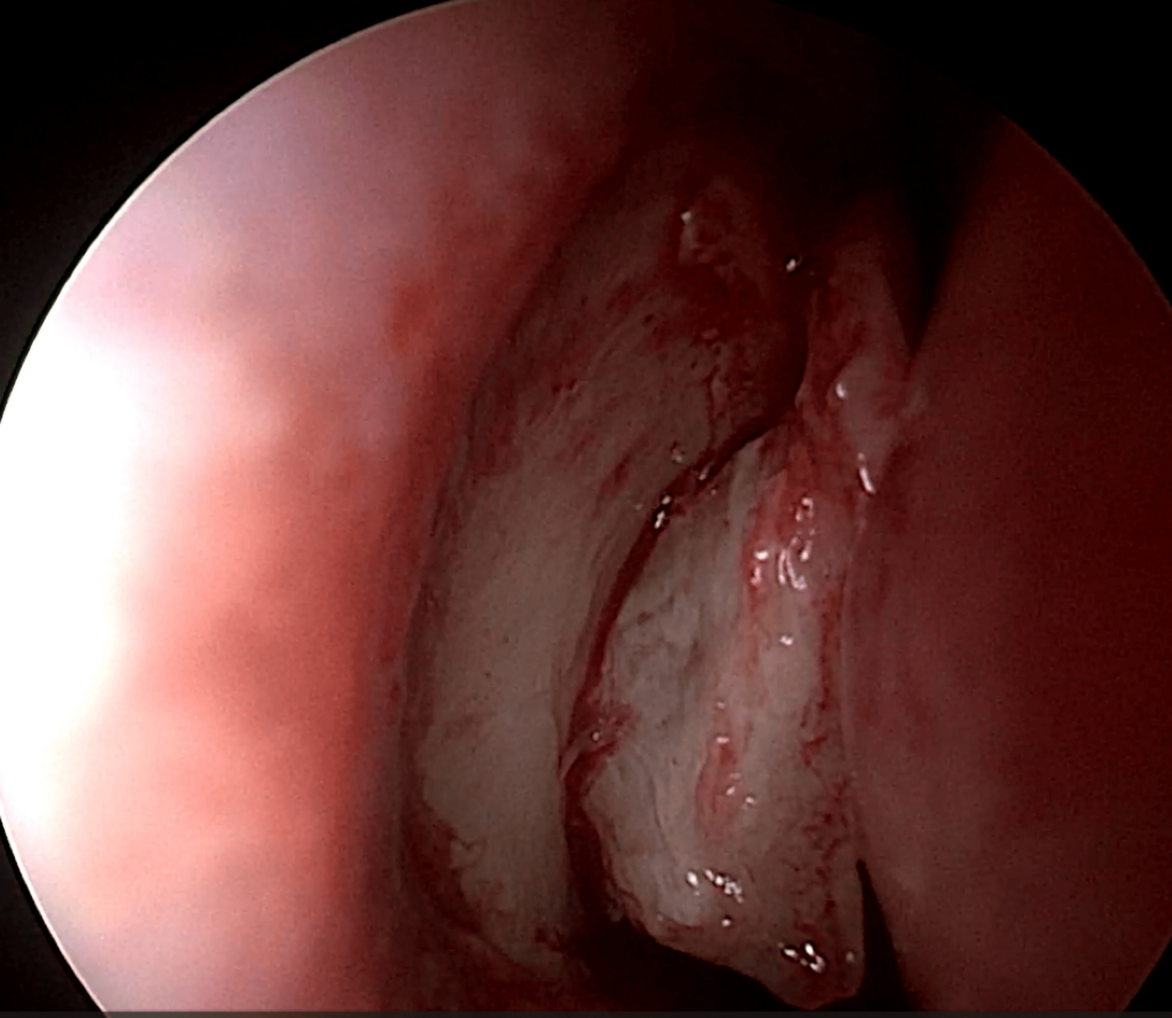
Mucoperiosteal flap elevated off frontal process of maxilla

**Figure 3:**
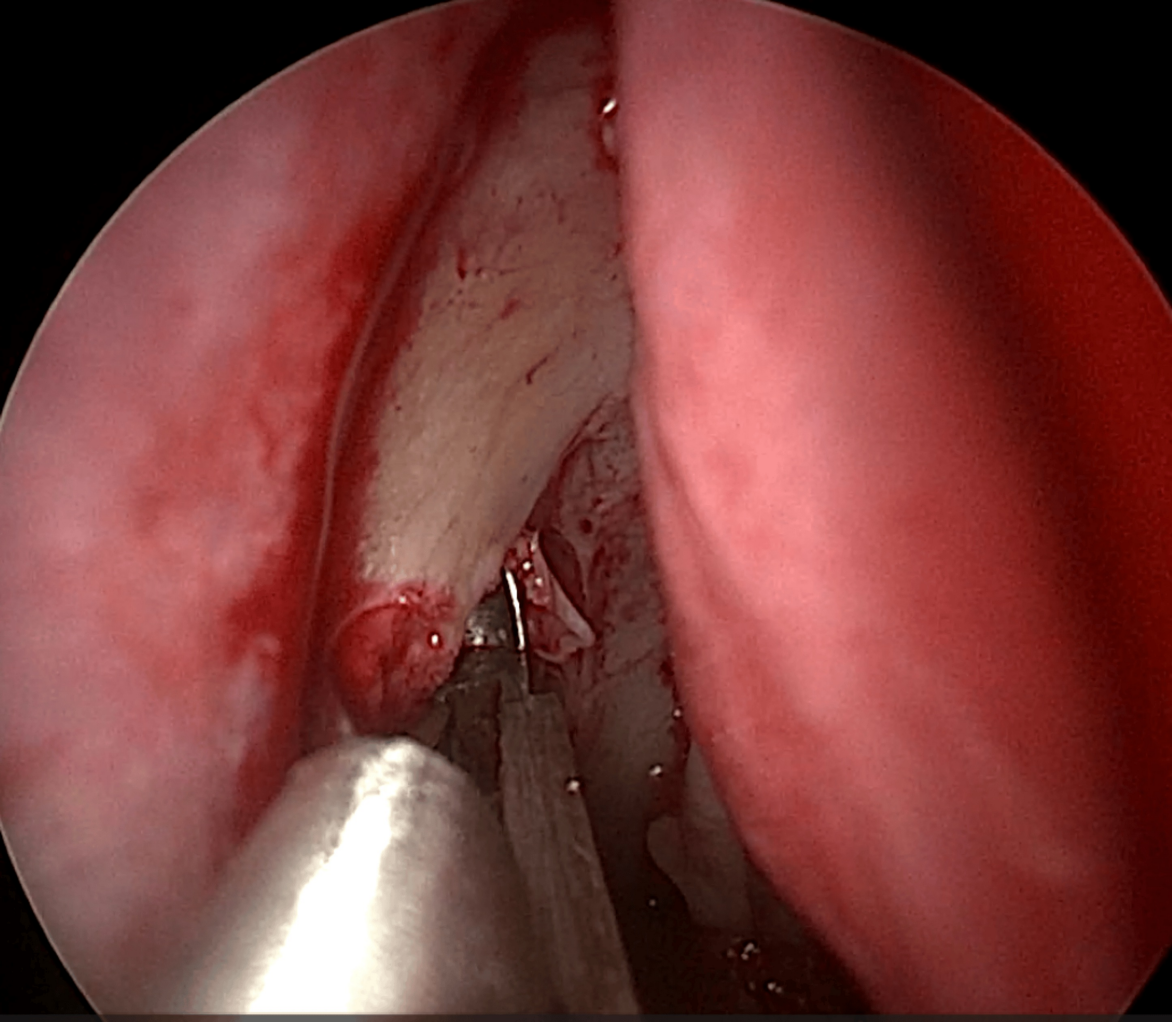
Endoscopic picture showing removal of bone over lacrimal sac using Kerrison punch

**Figure 4:**
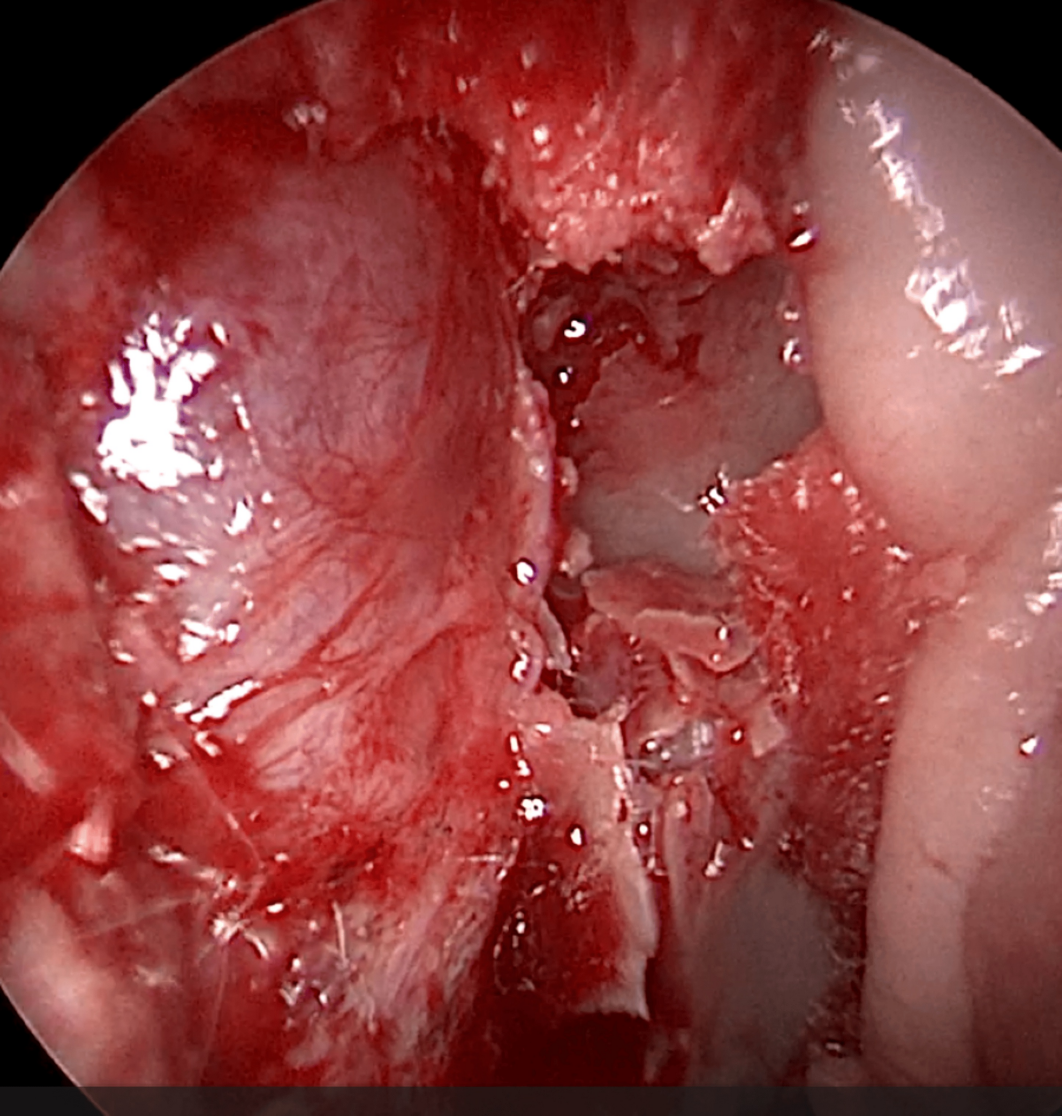
Endoscopic picture showing complete exposure of lacrimal sac

**Figure 5:**
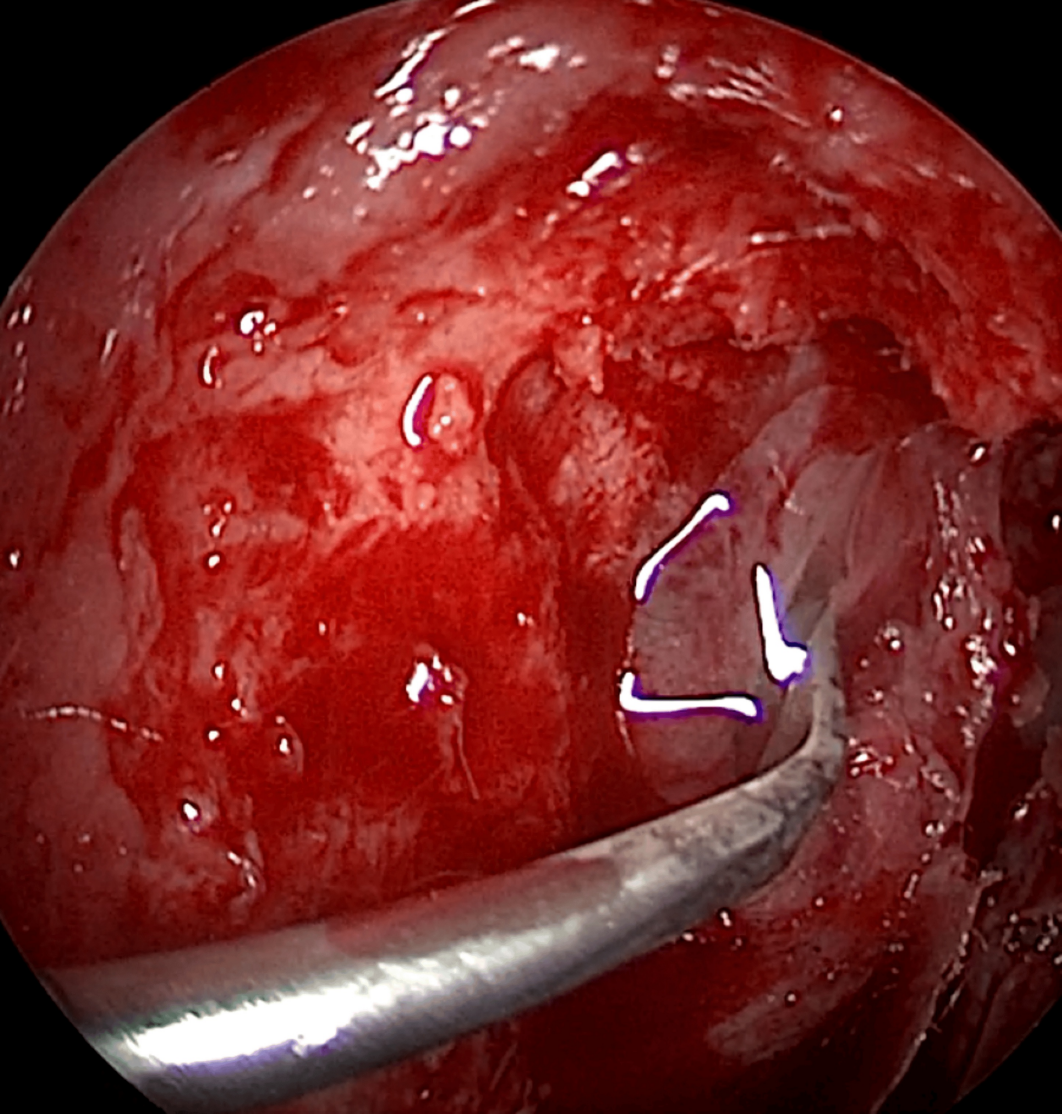
Incision of lacrimal sac with draining mucopus

**Figure 6:**
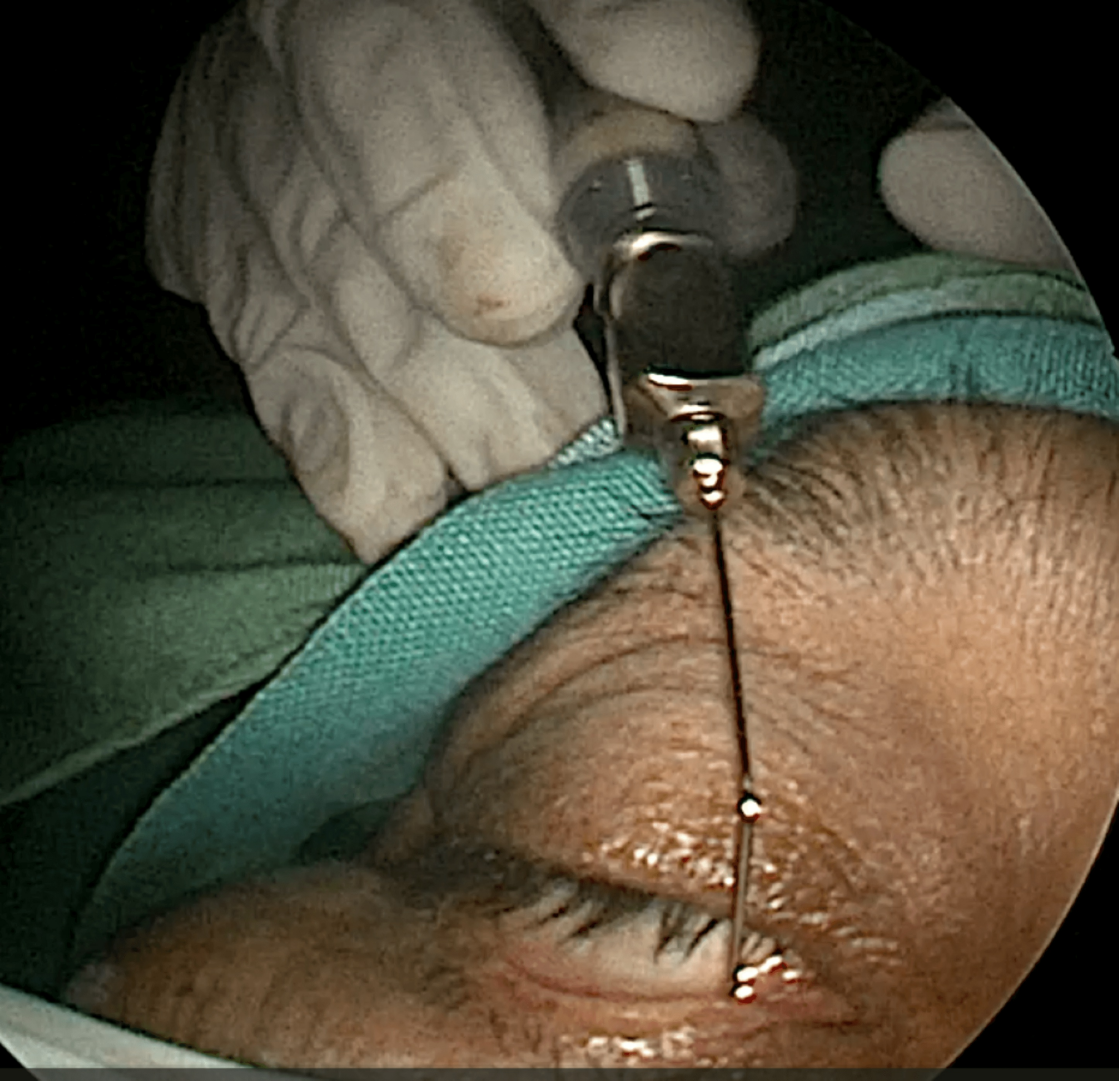
Intraoperative picture showing lacrimal syringing

**Figure 7:**
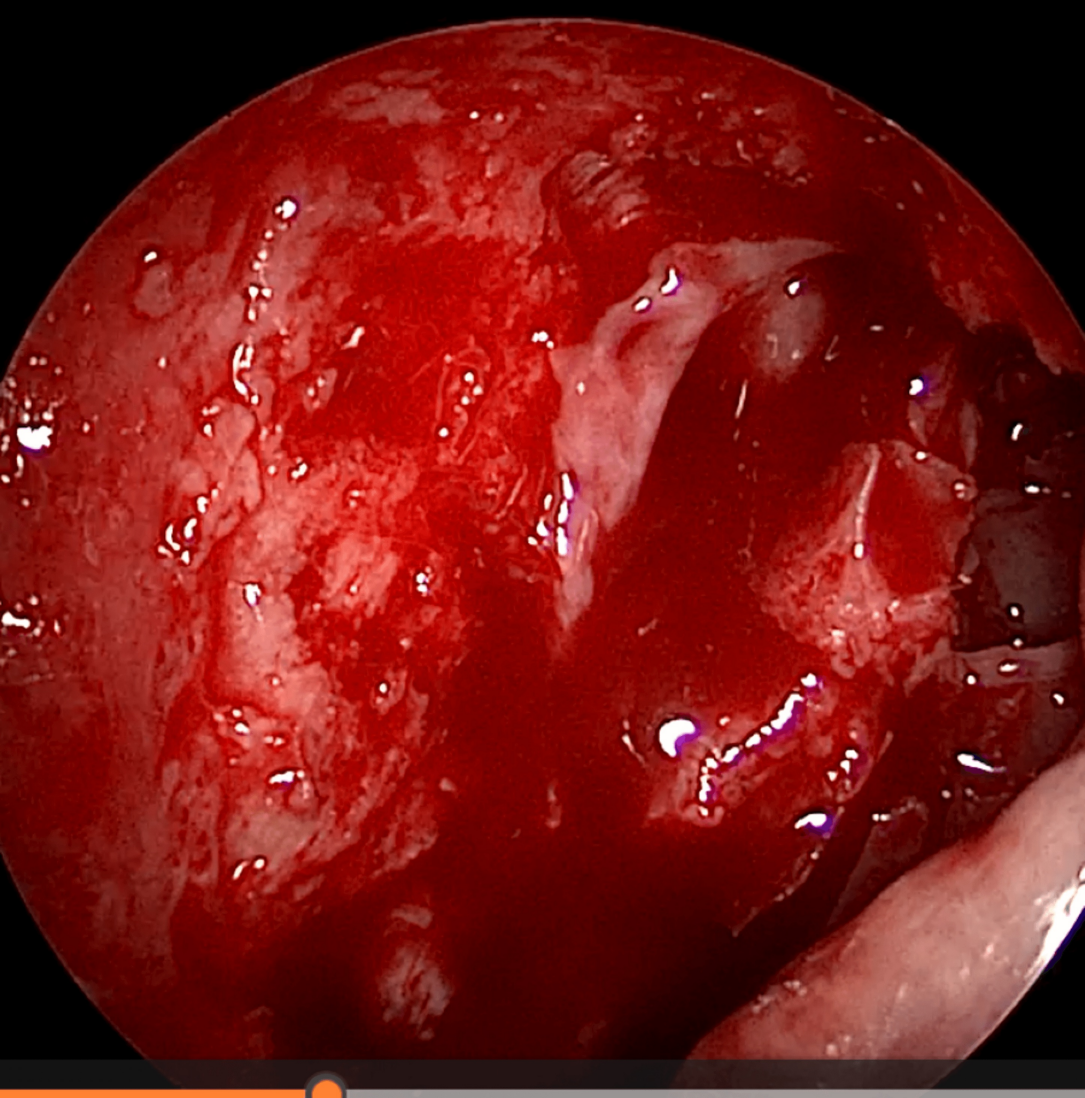
Marsupilisation of sac with anterior and posterior flaps

**Figure 8:**
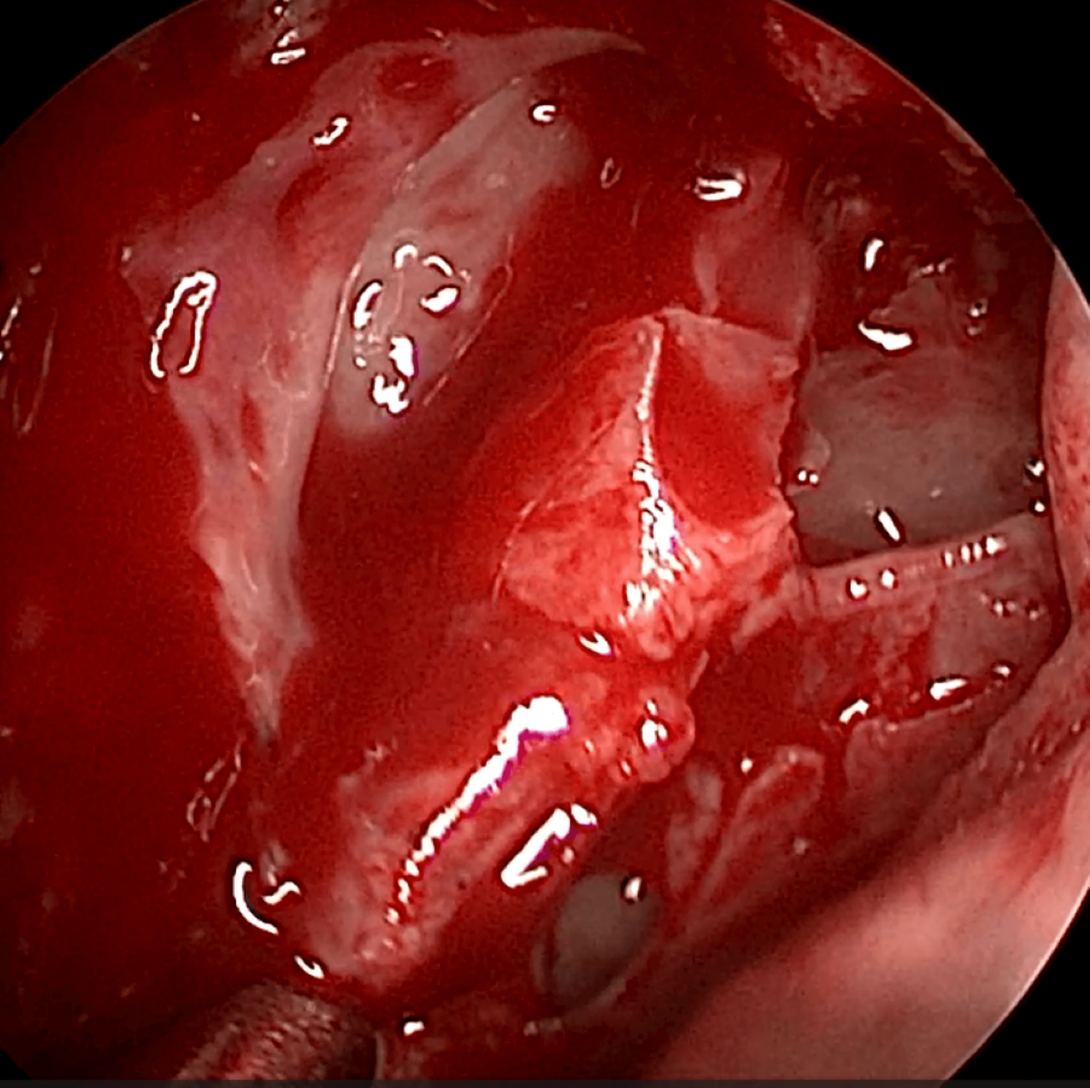
Marsupilisation of sac with anterior and posterior flaps

**Figure 9:**
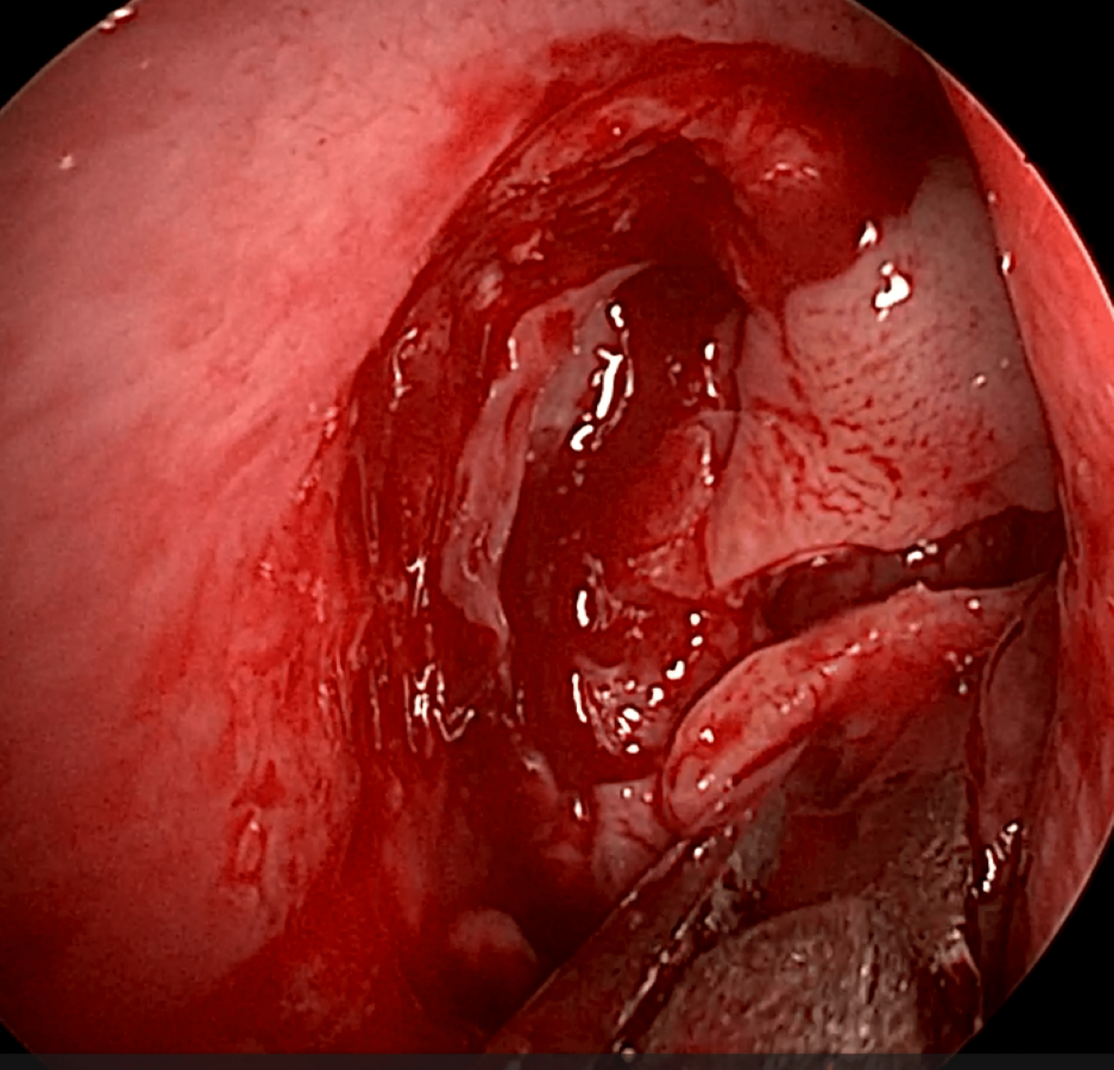
Mucoperiosteal flap trimmed and a rectangular mid segment excised to accomdate opened sac

**Figure 10:**
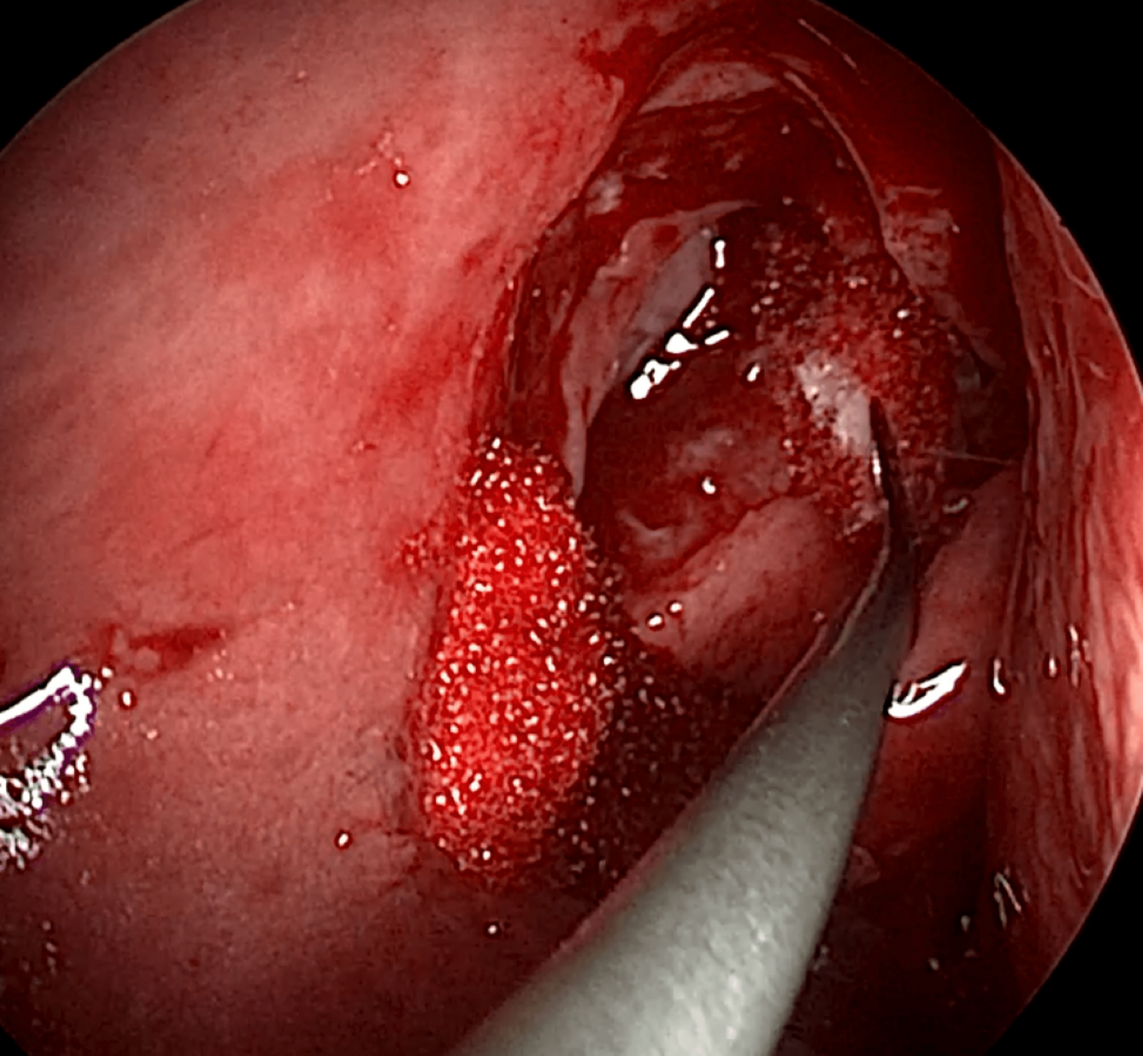
Reposited flap supported with gelfoam inferiorly and posterosuperiorly

**Figure 11:**
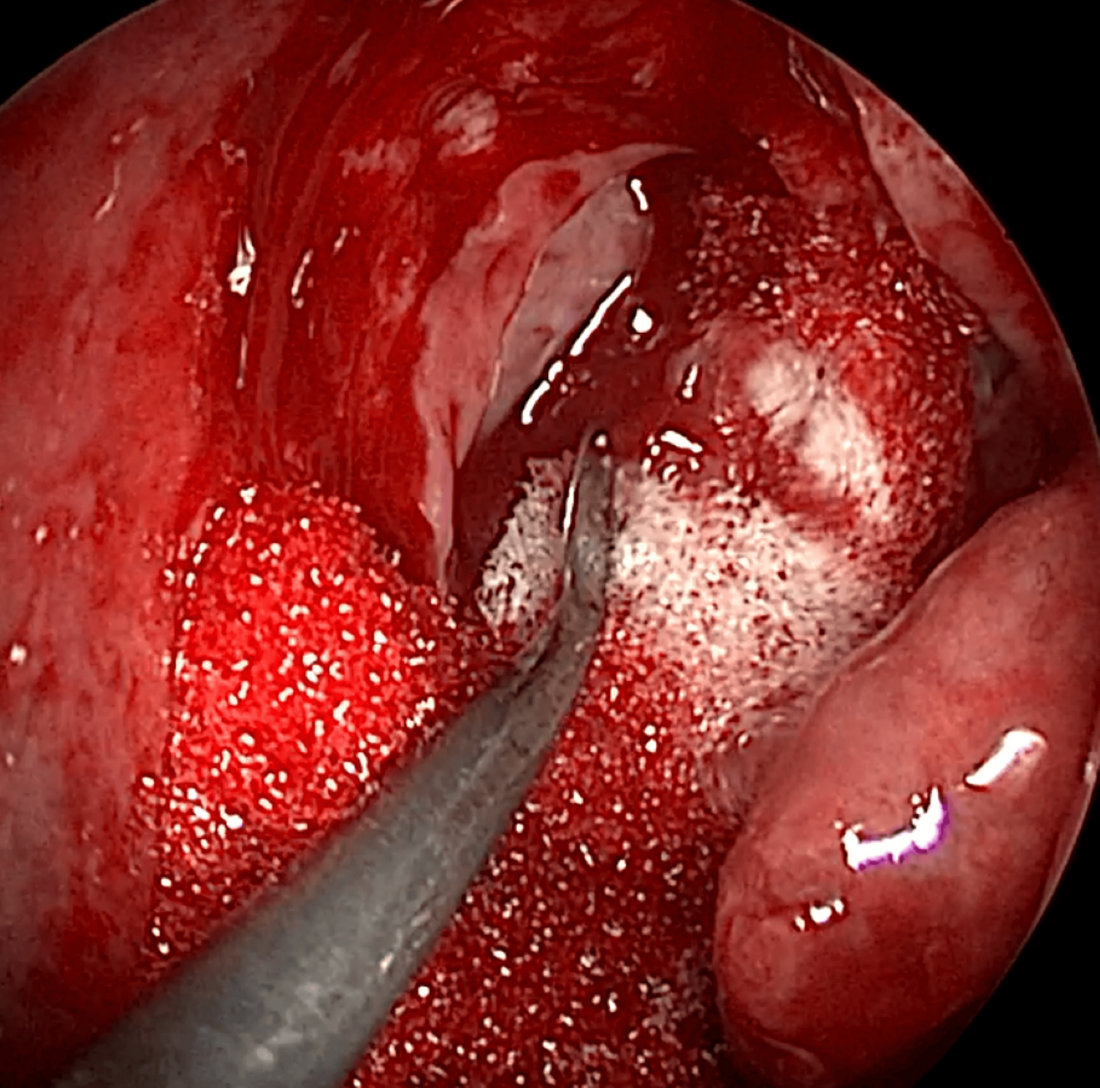
Reposited flap supported with gelfoam inferiorly and posteriorly

**Figure 12:**
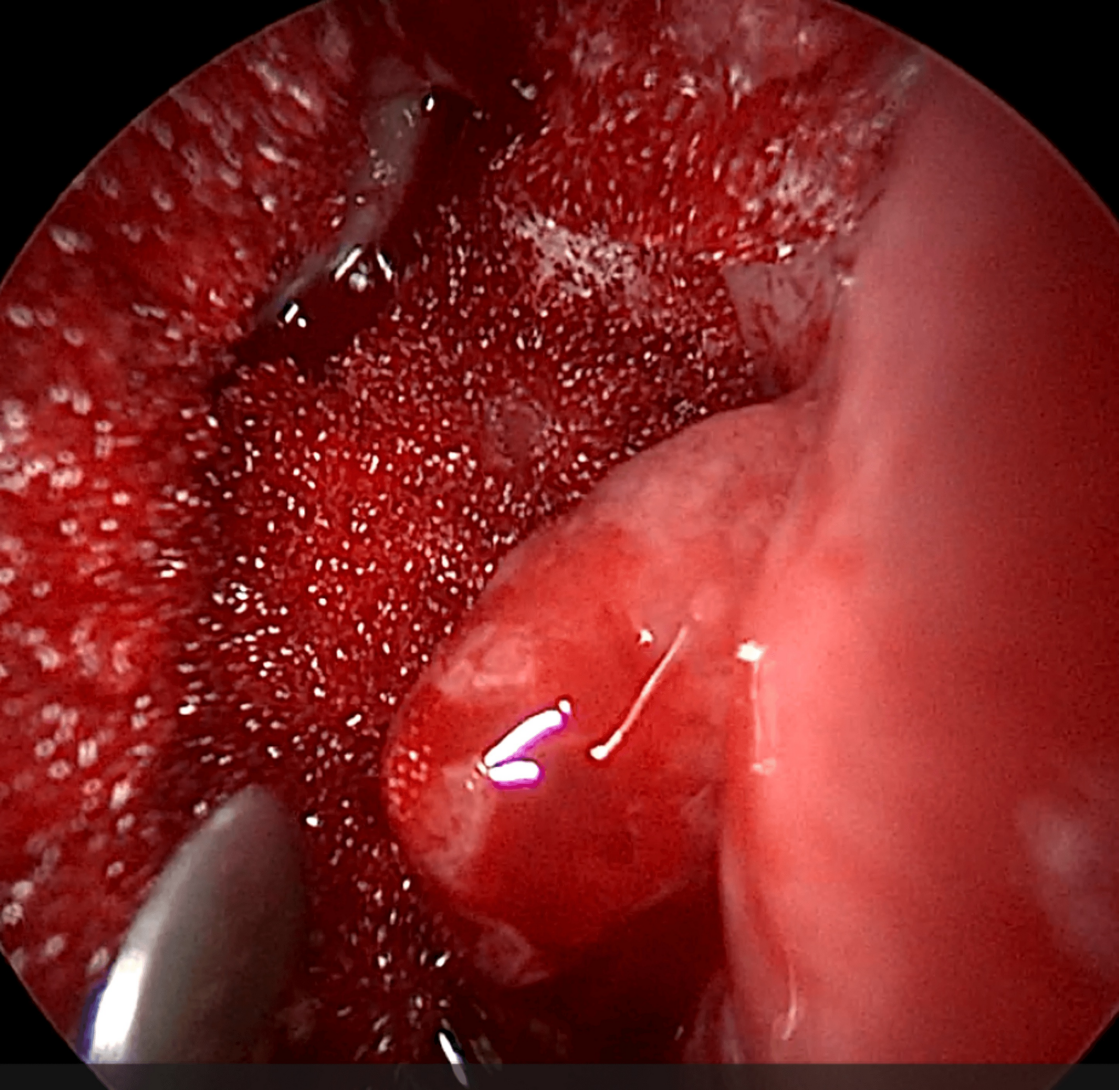
Reposited flap with sac supported with gelfoam all around

**Figure 13:**
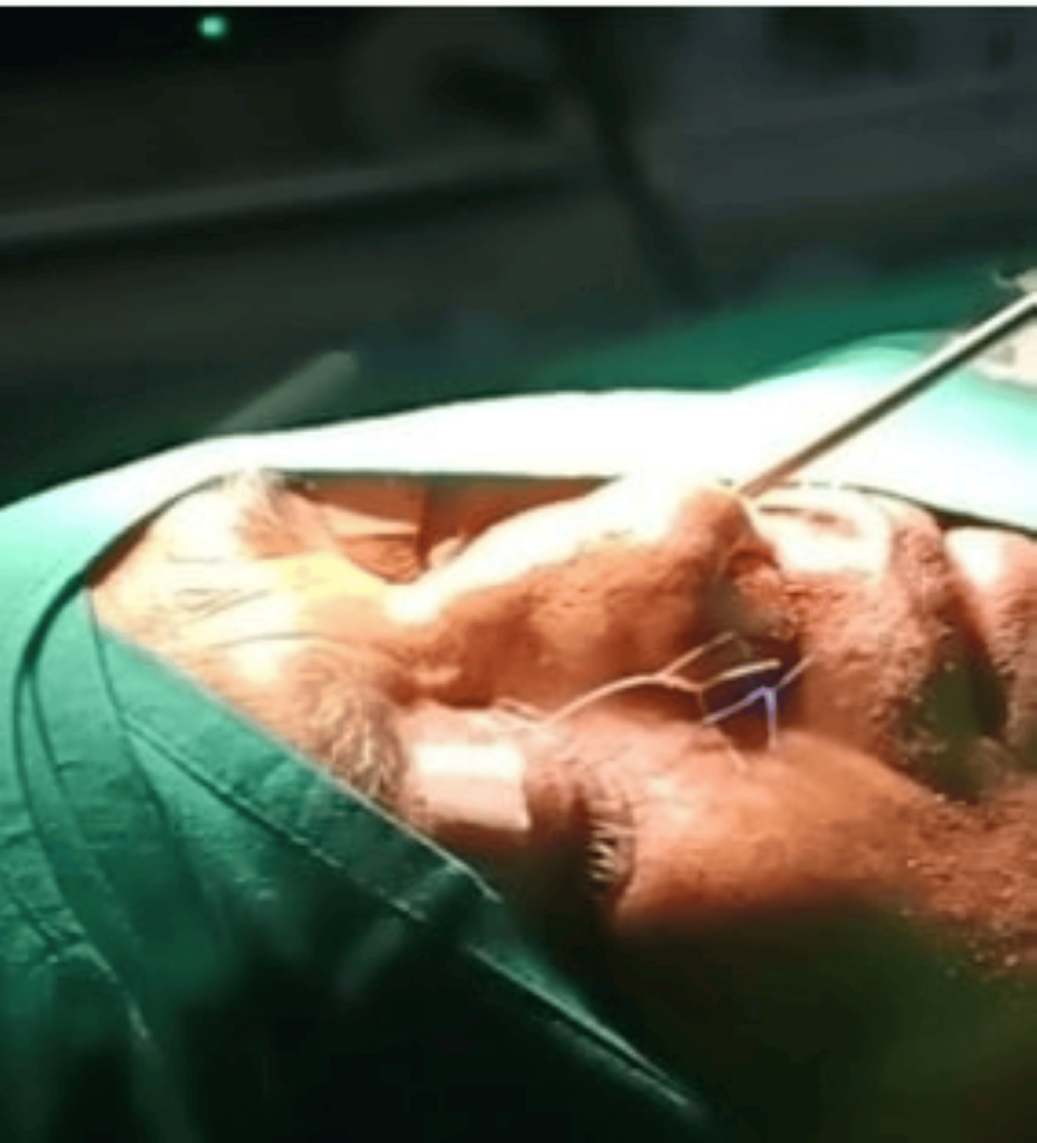
Intraoperative picture showing silicone stent and prolene suture stent insitu

EE-DCR may be performed under local or general anesthesia, with the choice determined by patient preference, clinical suitability, and tolerance to either modality. Nasal mucosal preparation is accomplished through the application of nasal packs imbued with a 4% solution of lignocaine and adrenaline at a dilution of 1:100,000. Infiltration of the lateral nasal wall was performed utilizing a solution of 2% lignocaine combined with 1:100,000 adrenaline. The patient is positioned in a supine orientation with the head elevated at an angle of 15 degrees. A 4-mm 0° endoscope was routinely used, while 30° and 70° endoscopes were additionally utilized when required to achieve optimal visualization of the entire sac. Two horizontal 1-cm incisions were made on the lateral wall of the nose, one at the level of approximately 5 mm above the axilla of the middle turbinate and another at the level of the superior margin of the inferior turbinate. Using a round knife, a vertical incision is made joining these two horizontal incisions (Figure 1). The mucoperiosteal flap is elevated backward off the maxillary bone (Figure 2), and the osseous covering of the lacrimal sac is then meticulously excised using Kerrison’s bone punch (Figure 3) until the sac is thoroughly exposed (Figure 4). A vertical incision is executed with a sickle knife or a keratome along the entire length of the exposed lacrimal sac (Figure 5). The sac is incised, and the patency of the ostium is assessed by syringing through the lacrimal puncta (Figure 6) or by introducing a Bowman’s lacrimal probe into it. The sac wall was meticulously crafted in a manner reminiscent of an open book (Figures 7, 8), with the flap edges precisely adjusted to align with the nasal mucosa (Figure 9), thereby ensuring unobstructed patency and further reinforced with gelfoam (Figures 10-12). In revision cases, silicone stents or 2-0 Prolene (Figure 13) are meticulously secured and retained for a duration of three months.

**Figure 14:**
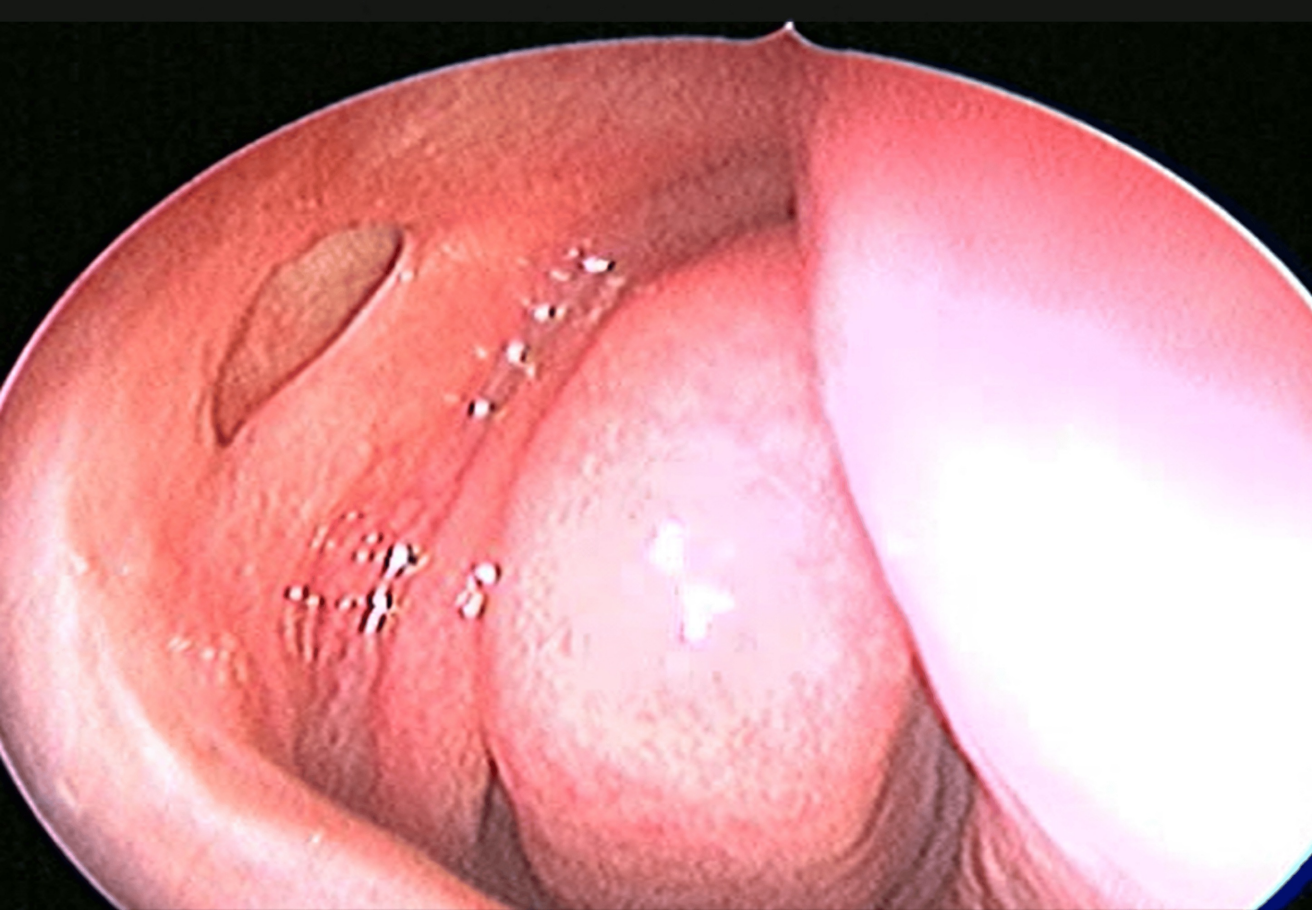
Postoperative endoscopic picture after six months

Postoperatively, all patients received a regimen of systemic antibiotics (Tab amoxycillin-clavulonic acid 25 mg/kg/dose) for seven days, anti-inflammatory medications (tablet composed of aceclofenac 100 mg, paracetamol 325 mg, trypsin-chymotrypsin 50,000 AU) for five days, antibiotic eye drops (0.5% moxifloxacin) one drop four times a day in the operated eye for two weeks, nasal decongestants (0.1% xylometazoline nasal drops) three drops three times a day for five days, and saline nasal spray two puffs three times a day for one month. They were also instructed to perform lacrimal massage three to five times daily. Follow-up consultations were scheduled weekly for the first month, subsequently monthly for the next three months, and finally at the six-month mark. At each consultation, the condition of the nasolacrimal duct was meticulously evaluated through syringing and nasal endoscopy, and patency was thoroughly examined (Figure 14).

A successful surgical outcome was defined by anatomical patency of the nasolacrimal duct, confirmed by lacrimal syringing and endoscopic visualization of the stoma, along with symptomatic relief reported by the patient. The assessment of symptomatic relief was conducted utilizing the Nasolacrimal Duct Obstruction Symptom Score (NLDO-SS) questionnaire graded from 0 (no symptoms) to 10 (worst imaginable symptom), which is a validated instrument for subjective postoperative outcome evaluation following EE-DCR [7]. Additionally, the quality of life before and after the surgical procedure was gauged using a distinct set of questionnaires [8], graded from 0 to 3, where 0 means not bothersome, 1 = mild, 2 = moderate, and 3 = severely affecting. Both assessments were performed at the conclusion of six months postoperatively. These data were entered into MS Excel 2019 version (Microsoft Corporation, Redmond, Washington) and further analyzed using IBM SPSS Statistics for Windows, Version 26 (Released 2019; IBM Corp., Armonk, New York). For descriptive analysis, the categorical variables were presented as frequency and percentages, and the continuous variables were presented as mean ± standard deviation or median and interquartile range as appropriate. The normality of data was tested with the Shapiro-Wilk test. The preoperative and postoperative quality-of-life scores were compared using a paired t-test or Wilcoxon signed-rank test, as appropriate. A p < 0.05 was considered statistically significant.

## Results

A total of 50 patients underwent surgery during the study period, comprising 19 (38%) males and 31 (62%) females. The ages of the participants ranged from 17 to 75 years, with the majority falling in the range 46-55 years (Table 1), and the mean age of the patients who underwent EE-DCR is 35.2 ± 13.3 years.

**Table 1:** Clinical profile of the patients

Clinical profile	No of patients	Percentage
Gender		
Female	31	62%
Male	19	38%
Age group		
16-25 years	2	4%
26-35 years	7	14%
36-45 years	12	24%
46-55 years	13	26%
56-65 years	10	20%
66-75 years	6	12%

Out of 50 surgical cases, 36 (72%) involved the right side, while 14 (28%) pertained to the left side. A significant 42 (84%) of the cases exhibited persistent epiphora from the affected eye, 28 (56%) patients experienced mucopurulent discharge from the lacrimal sac, 8 (16%) presented with mucocele, and 4 (8%) patients had pyocele. Septoplasty was performed in 12 (24%) cases to rectify a deviated nasal septum, thereby ensuring adequate exposure of the operative site. The mean duration of EE-DCR was 42 minutes (SD ± 23.7), whereas the procedure accompanied by septoplasty extended to 66 minutes (SD ± 25.0).

Anatomical success was attained in 48 (96%) patients, while subjective success was realized in 49 (98%) cases. The complications encountered included moderate hemorrhage during the surgical procedure in two patients (revision cases), postoperatively, granulation tissue at the stoma site in two patients, and nasal adhesions in two patients.

The mean preoperative symptom scores of the patients, as quantified using the NLDO-SS, were recorded at 22.8 ± 10.3. In contrast, the postoperative symptom scores, assessed at the conclusion of six months, were 3.96 ± 2.54. A noteworthy reduction in symptom scores was observed across all eight symptoms, accompanied by a statistically significant decrease in the total score (p < 0.001) (Table 2).

**Table 2:** Preoperative and postoperative NLDO-SS NLDO-SS: Nasolacrimal Duct Obstruction Symptom Score.

Symptom	Preoperative score, mean±SD	Postoperative score, mean±SD	t-value	Degree of freedom	P-value
Tearing	5.78±2.66	0.10±0.707	14.638	49	<0.001
Discharge in the eye	2.92±2.137	0.10±0.707	8.875	49	0.001
Swelling around the eye	1.60±1.77	0.04±0.283	6.024	49	<0.001
Pain around the eye	0.80±1.629	0.10±0.112	3.473	49	0.001
Change in visual acuity	5.78±2.66	2.54±1.809	11.308	49	<0.001
Nose blockage	0.12±0.435	0.02±0.141	1.528	49	0.133
Nasal cavity discharge	0.21±0.821	0.18±0.164	1.421	49	0.121
General condition	5.78±2.66	1.16±1.646	11.352	49	<0.001
Total score	22.78±10.39	3.96±2.539	14.592	49	<0.001

Similarly, there was a significant improvement in the postoperative score used for quality of life assessment (Table 3).

**Table 3:** Preoperative and postoperative scores in improvement of quality of life

Does your watery eye bother you with your	Preop grade, mean±SD	Postop grade, mean±SD	t-value	Degree of freedom	P-value
Sight?	2.16±0.781	0.42±0.581	14.762	49	<0.001
Reading?	1.94±0.767	0.26±0.565	16.671	49	0.001
Driving?	1.68±0.713	0.26±0.565	14.294	49	0.003
Mood?	1.74±0.751	0.32±0.587	14.924	49	<0.001
Work?	1.94±0.767	0.28±0.573	16.361	49	0.001
Does your watery eye become embarrassing?	1.96±0.781	0.24±0.573	16.671	49	<0.001

## Discussion

The last two decades have witnessed a surge in the popularity of minimally invasive surgical procedures across various surgical disciplines, and otorhinolaryngology is no exception. With the advent of rod lens telescopes and enhanced instrumentation, the surgical management of the lacrimal system is now being adeptly performed by otolaryngologists, leading to the adoption of EE-DCR as a preferred alternative to the traditional external DCR carried out by ophthalmologists.

In our study, the predominant demographic comprised 62% females, a finding that aligns with numerous other studies. Females exhibit a heightened susceptibility to chronic dacryocystitis, attributed to the constricted lumen of the nasolacrimal duct [9,10]. This female predominance in our study is corroborated by studies of Aslam et al. [11], wherein 85.8% of the study population consisted of females, while Rajan et al. [12] reported a proportion of 56% females within their cohort.

In our study, the most prevalent presenting complaint was epiphora, occurring in 84% of patients, while lacrimal abscesses were observed in merely 8% of cases. These were managed with systemic antibiotics (amoxicillin-clavulanic acid, 25 mg/kg/dose) for 14 days, anti-inflammatory medication (a combination of aceclofenac 100 mg, paracetamol 325 mg, and trypsin-chymotrypsin 50,000 AU) for seven days, and topical antibiotic eye drops (0.5% moxifloxacin), one drop four times daily for 14 days, followed by definitive management with EE-DCR. Furthermore, no patients exhibited lacrimal fistulae. Rajan et al. [12], in their investigation, noted that 88% of patients reported epiphora as the primary complaint, with 10% presenting with lacrimal abscess and a mere 2% having lacrimal fistula.

In our investigation, 24% of patients exhibited septal deviation on the affected side, necessitating septoplasty in conjunction with EE-DCR. The remaining 76% of patients underwent EE-DCR exclusively, with none receiving concurrent turbinectomy, polypectomy, or functional endoscopic sinus surgery (FESS). In the study conducted by Aslam et al. [11], EE-DCR accompanied by septoplasty was performed in 50.9% of the patients, whereas the research by Rajan et al. [12] revealed that only 8% of patients underwent septoplasty and 12% underwent FESS as simultaneous procedures.

As articulated by Tsirbar and Wormald [13], the cornerstone of successful EE-DCR lies in the meticulous exposure of the lacrimal sac and its subsequent marsupialization into the lateral nasal wall. This technique facilitates the apposition of the nasal and lacrimal mucosa, thereby promoting healing by primary intention, as opposed to the formation of granulation tissue. Such an approach significantly diminishes the risk of occlusion at the sac's orifice into the nasal cavity.

Our methodology encompasses the development of an extensive bony ostium and the formation of a posterior flap at the medial sac wall, which is subsequently retracted posteriorly and approximated with the nasal mucosal flap. Furthermore, a superior part of the posterior nasal mucosal flap is meticulously crafted to envelop the exposed osseous structure superiorly along the lateral nasal wall. Mann and Wormald [14] observed and articulated that the DCR ostium undergoes a slight yet significant reduction in size during the initial four weeks post-surgery, after which it stabilizes. The establishment of a substantial bony ostium and the complete exposure of the lacrimal sac are paramount for attaining both anatomical and subjective success.

During our study period, we encountered four revision cases, of which two involved failed external DCR, while the remaining two pertained to unsuccessful endonasal DCR procedures performed elsewhere. Among these four revision cases, two were successfully managed with silicone stents; however, the remaining two cases, which involved failed external DCR, presented significant challenges in securing the stents due to the presence of deep ocular sockets, altered anatomy resulting from previous surgical interventions, and a fractured stent during passage through the upper punctum. To address this complication, we employed an innovative technique wherein 2-0 Prolene suture material was meticulously threaded separately through the upper punctum into the nasal cavity using 22-gauge microear suction tips in both patients. Subsequently, two external loops were fashioned and left in situ for a duration of three months. Patients were instructed to rotate the loops intermittently on a daily basis. Remarkably, this technique facilitated the attainment of patency during lacrimal syringing, and both patients reported complete resolution of symptoms after three months. Notably, in one patient, patency was achieved exclusively through the upper punctum; however, regurgitation was observed through the lower punctum.

In our study, by the end of six months, anatomical success was observed in 96% of patients, while subjective success was achieved in 98%. Aslam et al. [11] reported an anatomical success rate of 96.2% and a subjective success rate of 95.35% at eight months. Rajan et al. [12] documented a success rate of 94% at two years, whereas Majumder et al. [13], using a similar mucosal flap technique, reported a success rate of 89% at 6-12 months.

The intraoperative complications associated with EE-DCR may encompass hemorrhage, orbital injury, hematoma formation within the lamina papyracea, and, in rare instances, endophthalmitis. Nonetheless, at our institution, only intraoperative hemorrhage was documented in two patients; apart from this, no other significant intraoperative complications were encountered. In the two instances that experienced intraoperative hemorrhage, characterized as moderate in severity, we utilized the Boezaart score to assess the surgical field [15,16]. In our cases, intraoperative hemorrhage was adeptly managed through a multifaceted approach: abundant cold saline irrigation, nasal packing with adrenaline-soaked gauze for no less than five minutes, and the collaborative assistance of the anesthesiologist to facilitate controlled hypotension. These interventions ensured a clear surgical field and significantly mitigated intraoperative blood loss. Postoperatively, two patients exhibited granulation tissue at the ostium site, while another two developed adhesions either between the middle turbinate and the lateral nasal wall or between the lateral wall of the nose and the septum.

EE-DCR, in contrast to the conventional external approach, offers numerous advantages, such as the elimination of facial scarring, preservation of lacrimal pump function, and a more efficient procedural duration [17].

## Conclusions

In this contemporary era characterized by the ubiquitous availability of endoscopes, endonasal endoscopic DCR emerges as a markedly superior alternative to traditional external DCR. This technique obviates the formation of external scars, preserves the functional integrity of the orbicularis oculi muscle, and facilitates the concurrent management of associated sinonasal pathologies, thereby promoting an accelerated postoperative recovery. This study meticulously evaluated both the amelioration of symptoms following surgery and the enhancement of quality of life subsequent to EE-DCR intervention. The principal limitation of this investigation lies in its modest sample size and retrospective design. Moreover, the postoperative follow-up period was confined to six months.
